# Supplementary material for: Efficient two-step chemoenzymatic conjugation of antibody fragments with reporter compounds by a specific thiol-PEG-amine Linker, HS-PEG-NH2
Source: PLoS One. 2025 Oct 23;20(10):e0333359. doi: 10.1371/journal.pone.0333359 (PMC12548897; doi:10.1371/journal.pone.0333359)
Supplement: S1 Text — (PDF) [file pone.0333359.s001.pdf]

Supplementary Materials and Methods

Construction of Fab$_{p24}$ and Fab$_{CD20}$ expression vectors. Fab$_{p24}$ is a heterodimer composed of light (L) and heavy (H) chains linked by an S–S bond, with the carboxyl terminus of the constant region of the H chain fused to the original Q1 tag, which contains the sequence GVLNLAQSP [1]. The 20 residues, including the terminus with the tag, were VDKKVEPKHSCGVLNLAQSP. The average molecular masses of the L and H chains, calculated from their sequences, were 24897.874 and 23593.337 Da, respectively. Fab$_{p24}$ was generated in our laboratory. Briefly, female BALB/c mice (Japan SLC) at 5 weeks of age were immunized with the recombinant HIV-1 p24 protein. Approval was received from the institutional animal ethics committee of Sysmex Corporation. Lymphocytes were fused with mouse myeloma using the polyethylene glycol method [2], and hybridomas secreting anti-HIV-1 p24 antibody was created. The variable regions of L and H chain cDNAs were obtained from the hybridomas and were ligated to the constant region of the human immunoglobulin (Ig) L chain kappa and the IgG1 CH1 domain fused with the Q1 tag, respectively. A signal peptide sequence was attached to the 5' end of each cDNA and inserted downstream of the CMV promoter in the pcDNA3.4 expression vector (Thermo Fisher Scientific, USA).

For Fab$_{CD20}$, cDNA encoding the amino acid sequences below was chemically synthesized and inserted downstream of the CMV promoter in the pcDNA3.4 expression vector. The amino acid sequences of the chain are as follows:

L chain,

QIVLSQSPAILSASPGEKVTMTCRASSSVSYIHWFQQKPGSSPKPWIYATSNLASGVPVRF

SGSGSGTSYSLTISRVEAEDAATYYCQQWTSNPPTFGGGTKLEIKRTVAAPSVFIFPPSDE
QLKSGTASVVCLLNNFYPREAKVQWKVDNALQSGNSQESVTEQDSKDSTYSLSSTLTLS
KADYEKHKVYACEVTHQGLSSPVTKSFNRGEC

H chain,

QVQLQQPGAELVKPGASVKMSCKASGYTFTSYNMHWVKQTPGRGLEWIGAIYPGNGD
TSYNQKFKGKATLTADKSSSTAYMQLSSLTSEDSAVYYCARSTYYGGDWYFNVWGAG
TTVTVSAASTKGPSVFPLAPSSKSTSGGTAALGCLVKDYFPEPVTVSWNSGALTSGVHTF
PAVLQSSGLYSLSSVVTVPSSSLGTQTYICNVNHKPSNTKVDKKAEPKSCDKTHTGLLQ
G

These sequences correspond to the L and H chains of the chimeric anti-CD20 antibody rituximab [3–5], with the Q2 tag, GLLQG [6–8], fused to the carboxy terminus of the H chain.

Expression and purification of recombinant Q-tagged Fabs. Recombinant Q-tagged Fab, Fab_{p24}, or $Fab_{CD20,}$ was expressed using the Expi293 expression system (Thermo Fisher Scientific, USA) according to the manufacturer's protocol [9]. The pcDNA3.4 vector-transfected 293F cells were cultured at 37 °C, in a humidified atmosphere with 8% CO_2 and rotation at 125 rpm. After 7 days of post-transfection, cells were removed by centrifugation at 20,000 × g for 20 min, and the supernatant was filtered through a 0.2 μm sterile filter (Minisart, Germany). The supernatant was applied directly to a HiTrap Protein G HP column (1 mL; Cytiva, USA) equilibrated with 20 mM sodium phosphate buffer (pH 7.0). After washing the column with 20 mM sodium phosphate buffer, the Fab was eluted with a 0.1 M glycine solution (pH 2.7). Immediately after neutralization with 1 M Tris buffer (pH 8.5), the Fab was dialyzed overnight against PBS (-) (pH 7.4) at 5 °C and stored at −80 °C until use.

Fab_{p24} has been confirmed to be specific for HIV-1 p24 by the WHO International Reference Panel for HIV-1 P24 Antigen (1st international reference panel) 16/210. Furthermore, the background of the Fab_{p24} ELISA assay was corrected by a spike/recovery test [10] with human serum specimens spiked with p24 antigen.

Pentylamine-biotin modification of Q-tagged Fab by MTGase. Q-tagged Fab_{p24} was conjugated with 5-(biotinamido)pentylamine (pentylamine-biotin; MW; 328.48; Molecular Formula: $C_{15}H_{28}N_4O_2S$; Thermo Fisher Scientific) using MTGase. The reaction was performed in 20 mM Tris/HCl buffer containing 150 mM NaCl (pH 7.5) at 37 °C for 4 h with the following reagent concentrations: Q-tagged Fab_{p24} (10 μM), pentylamine-biotin (500 μM), and MTGase (0.1U/mL) [1]. The product was confirmed by HIC-HPLC (see below), purified by analytical SEC, dialyzed against PBS (II) overnight at 5 °C, and stored at 5 °C before use. Biotin incorporation was confirmed by western blotting following the method described by Pitek et al. [11], using horseradish peroxidase (HRP)-conjugated streptavidin (Thermo Fisher Scientific, USA).

Hydrophobic interaction chromatography-HPLC analysis. The HPLC was performed using Chromaster™ (Hitachi High-Tech Science Corporation, Japan) with a PolyPROPYL A column (100 × 4.6 mm, Polylc Inc., USA). The mobile phases composed of A and B consisted of 1.5 M ammonium sulfate and 35% acetonitrile in 20 mM sodium phosphate buffer (pH 7.0), respectively. Gradient elution was performed from 0% to 50% B for 15 min and then back to 0% B for a further 7 min at a flow rate of 0.8 mL/min at room temperature. Approximately 5 μg of the sample protein was loaded into the column, and the absorbance was recorded at 280 nm.

LC-MS/MS analysis of tryptic digest of Fab. For LC-MS/MS analysis, 2 μL of 1 M Tris-HCl solution (pH 8.5) was added to 20 μL of 0.25 mg/mL Fab solution in milli-Q H_2O containing 8 M urea (for Molecular Biology, FUJIFILM Wako Pure Chemical Corporation). After adding 1 μL of 0.1 M DTT under argon, the solution was incubated for 1 h at room temperature. Subsequently, 1 μL of 0.2 M iodoacetamide (biochemical grade; Fujifilm Wako Pure Chemical Corporation, Japan) was added to the solution, and the sample was incubated for 1 h at room temperature in the dark under an argon atmosphere. After diluting the sample by adding 60 μL of milli-Q H_2O, 1 μL of trypsin solution (Sequencing grade modified Trypsin, Promega, 1 mg/mL) was added and incubated for 12–14 h at 37 °C. The pH of the digest was adjusted to less than 3 using formic acid for column chromatography (Nacalai Tesque, Inc., Japan). The supernatant (20 μL) was transferred to an autosampler vial for LC-MS/MS analysis.

LC-MS and LC-MS/MS analyses were performed using a Develosil C18HG reversed-phase column (2.0 mm I.D. × 100 mm, 3 μm particle size; Nomura Chemical, Japan) connected to a Q Exactive mass spectrometer (Thermo Fisher Scientific) via an electrospray interface. Samples were eluted with a 30-min linear gradient of 5%–45% acetonitrile in 0.1% formic acid, followed by 10 min at 45% acetonitrile and 10 min at 70% acetonitrile, at a flow rate of 100 μL/min. The eluate from the first 6 min was discarded by switching a three-way electric valve to remove urea from the system before introduction into a mass spectrometer. Mass spectra were acquired at 3.0 kV in the positive mode at a resolution of 35,000 for m/z 300–1,500 for LC-MS and 17,500 for m/z 200–2,000 for LC-MS/MS.

Supplementary Results

Characterization of 3.5 and 5 kDa HS-PEG linkers and their reaction efficiency. The HS-PEG$_{3.5k}$ and HS-PEG$_{5k}$ linkers (Sigma-Aldrich) were analyzed using direct infusion MS 30 min after dissolution (Figure S1). Details of the results are provided in the main text. Conjugation experiments with Fab and the HS-PEG$_{3.5k}$ and HS-PEG$_{5k}$ linkers were also performed, and the products were analyzed by analytical SEC (Figure S4). The reaction products of Fab$_{p24}$ with HS-PEG linkers showed an estimated reaction efficiency of approximately 95% based on peak areas. This efficiency was very high comparable to that of the HS-PEG$_{2k}$ linker.

Identification of modified residues in recombinant Q-tagged Fab by MTGase. To identify the residues modified by MTGase in Q-tagged Fab, we first modified Fab$_{p24}$ with pentylamine-biotin. As shown in Figure S2A, peak A of Fab$_{p24}$ disappeared after the reaction, and a new peak B appeared in the reaction product, indicating complete modification of Fab by pentylamine-biotin under these conditions. Both SDS-PAGE and Western blot analysis (Figure S2B) showed bands of approximately the same molecular weight, confirming that the major portion of peak B was biotinylated Fab$_{p24}$.

Next, we performed peptide mapping of Fab$_{p24}$ using LC-MS/MS. If pentylamine-biotinylated Fab$_{p24}$ is carbamoylmethylated and cleaved with trypsin, the Q-tagged peptide, SCGVLNLAQSP, contains Cys and Gln modified with a carbamoylmethyl residue and pentylamine-biotin, respectively, with a divalent cation at m/z 728.865. Therefore, we analyzed the trypsin digestion of Fab$_{p24}$ using an LC-MS/MS system (Figure S3), and the extracted ion chromatogram at m/z 728.865 (±20 ppm) revealed a peak corresponding to a peptide ion detected only in the biotinylated Fab$_{p24}$ sample (Figure S3D). Additionally, the MS/MS spectrum

of the peptide ion (m/z 728.87) showed that pentylamine-biotin was located at the Gln position within the sequence (Table S1). These results indicated that MTGase specifically modified the Gln in the Q1 tag.

To confirm that pentylamine-biotin modification by MTGase occurred exclusively at the Q tag within the entire Fab_{p24} sequence, we examined the product ions specific to pentylamine-biotin at m/z 329.2 and 395.2 [12]. Figure S3E shows the extracted chromatogram of the product ion at m/z 329.2, where the major peak was consistent with that shown in Figure S3D, and the m/z of the precursor ion matched that of the pentylamine-biotinylated Q-tag-derived peptide (m/z 728.87). Similar results were obtained when an m/z of 395.2 was used as the product ion (data not shown). These findings confirm that MTGase modification occurred at the Q-tag sequence. In combination with the MS/MS sequence analysis of the peptide (Table S1), we concluded that only a specific Gln residue in the Q-tag sequence of Fab_{p24} was modified by MTGase with pentylamine-biotin.

SPR analyses of Fab_{p24}, HS-PEG_{2k}-Fab_{p24}, and Alexa488-PEG-Fab_{p24} against HIV-1 p24 protein.

Fab and its derivatives were immobilized on a sensor chip and injected at five different concentrations of recombinant HIV-1 p24 protein (Figure S5). As expected, HIV-1 p24 protein bound specifically to the Fabs. In contrast, no affinity for HIV-1 p24 protein was observed on sensor chips without the Fab (data not shown). The sensorgram profiles of HS-PEG_{2k}-Fab_{p24} and Fab_{p24} (Figure S5A and S5B) and Alexa488-PEG_{2k}-Fab_{p24} and Fab_{p24} (Figure S5C and S5D), each evaluated in a single batch of experiments, were highly similar, and their KDs were

equivalent. Based on these results, the antigen-binding activity of modified Fab_{p24} and Fab_{p24} were considered equivalent.

Separation of reaction products of Fab_{p24}, HS-PEG_{2k} linker, and maleimide-activated PE. The reaction product was separated using preparative SEC (Figure S6).

Details of reaction process of Fab_{CD20}, HS-PEG_{2k} linker, and maleimide-activated PE. We further examined the reactivity of different Fabs fused with different Q tags (Q2 and GLLQG) at the C-terminus of the H chain (Figure S7). Purified Fab_{CD20} (Figure S7A) containing the Q2 tag was subjected to a two-step reaction with the HS-PEG_{2k} linker and maleimide-activated PE. As shown in Figure S7B, in the presence of MTGase and the HS-PEG linker, Fab_{CD20} (peak A') decreased and a new peak B' appeared, indicating that the HS-PEG_{2k} linker was incorporated. The reaction efficiency of the MTGase-catalyzed modification was ~77%. Following the two-step reaction, including the purification and desalting (see Methods section in the main text), the yield of HS-PEG_{2k}-modified Fab_{CD20} (HS-PEG_{2k}-Fab_{CD20}) was 0.35 mg (approximately 41%), based on the starting material (0.85 mg).

This ~77% reaction efficiency of the Q2 tag fused Fab_{CD20} was unexpected because the reaction efficiencies of chemically synthesized peptides of several residues with the Q1 and Q2 sequences and pentylamine-biotin were similar (data not shown). We speculate the lower catalytic efficiency of MTGase for Q2 tag-fused Fab_{CD20} compared to that for Q1 tag-fused Fab_{p24} (>95%) is due to the proteolytic cleavage of the C-terminus of the Q2 tag (GLLQG-COOH) of Fab_{CD20}

during expression and purification (data not shown). This issue could be mitigated by adding a highly exopeptidase-resistant proline at the C-terminus.

References

1. Sato H, Hayashi E, Yamada N, Yatagai M, Takahara Y. Further studies on the site-specific protein modification by microbial transglutaminase. Bioconjug Chem. 2001;12: 701–710. doi:10.1021/bc000132h

2. Gefter ML, Margulies DH, Scharff MD. A simple method for polyethylene glycol-promoted hybridization of mouse myeloma cells. Somatic Cell Genet. 1977;3: 231–236. doi:10.1007/BF01551818

3. Reff M, Carner K, Chambers K, Chinn P, Leonard J, Raab R, et al. Depletion of B cells in vivo by a chimeric mouse human monoclonal antibody to CD20. Blood. 1994;83: 435–445. doi:10.1182/blood.V83.2.435.435

4. Du J, Wang H, Zhong C, Peng B, Zhang M, Li B, et al. Crystal structure of chimeric antibody C2H7 Fab in complex with a CD20 peptide. Mol Immunol. 2008;45: 2861–2868. doi:10.1016/j.molimm.2008.01.034

5. Nebija D, Kopelent-Frank H, Urban E, Noe CR, Lachmann B. Comparison of two-dimensional gel electrophoresis patterns and MALDI-TOF MS analysis of therapeutic recombinant monoclonal antibodies trastuzumab and rituximab. J Pharm Biomed Anal. 2011;56: 684–691. doi:10.1016/j.jpba.2011.07.006

6. Strop P, Liu SH, Dorywalska M, Delaria K, Dushin RG, Tran TT, et al. Location matters: Site of conjugation modulates stability and pharmacokinetics of antibody drug conjugates. Chem Biol. 2013;20: 161–167. doi:10.1016/j.chembiol.2013.01.010

7. Schneider H, Deweid L, Avrutina O, Kolmar H. Recent progress in transglutaminase-mediated assembly of antibody-drug conjugates. Anal Biochem. 2020;595: 113615. doi:10.1016/j.ab.2020.113615

8. Tanaka Y, Tsuruda Y, Nishi M, Kamiya N, Goto M. Exploring enzymatic catalysis at a solid surface: a case study with transglutaminase-mediated protein immobilization. Org Biomol Chem. 2007;5: 1764. doi:10.1039/b701595j

9. Expi293 Expression System [Internet]. [Cited 2017 Jun 13]. Available from: http://www.thermofisher.com/ug/en/home/life-science/protein-biology/ protein-expression/mammalian-protein-expression/transient-mammalianprotein-expression/expi293-expression-system.html.html?gclid= Cj0KEQjwmv7JBRDXkMWW4_ Tf8ZoBEiQA11B2fqYbB31lW1La5FohMYdPeobx4H0WVv_ xmrRNs4SlHMIaAgSb8P8HAQ&ef_id=WFBkEQAABM@NpM6E: 20170613124108:s.

10. Nakatsuma A, Kaneda M, Kodama H, Morikawa M, Watabe S, Nakaishi K, et al. Detection of HIV-1 p24 at attomole level by ultrasensitive ELISA with Thio-NAD cycling. PLoS One. 2015;10. doi:10.1371/journal.pone.0131319

11. Pitek AS, Wang Y, Gulati S, Gao H, Stewart PL, Simon DI, et al. Elongated Plant Virus-Based Nanoparticles for Enhanced Delivery of Thrombolytic Therapies. Mol Pharm. 2017;14: 3815–3823. doi:10.1021/acs.molpharmaceut.7b00559

12. Biberoglu K, Schopfer LM, Tacal O, Lockridge O. Characteristic fragment ions associated with dansyl cadaverine and biotin cadaverine adducts on glutamine. Anal Biochem. 2020;600: 113718. doi:10.1016/j.ab.2020.113718
