# Supplementary figures and images for: Efficient two-step chemoenzymatic conjugation of antibody fragments with reporter compounds by a specific thiol-PEG-amine Linker, HS-PEG-NH2
Source: PLoS One. 2025 Oct 23;20(10):e0333359. doi: 10.1371/journal.pone.0333359 (PMC12548897; doi:10.1371/journal.pone.0333359)

(A)

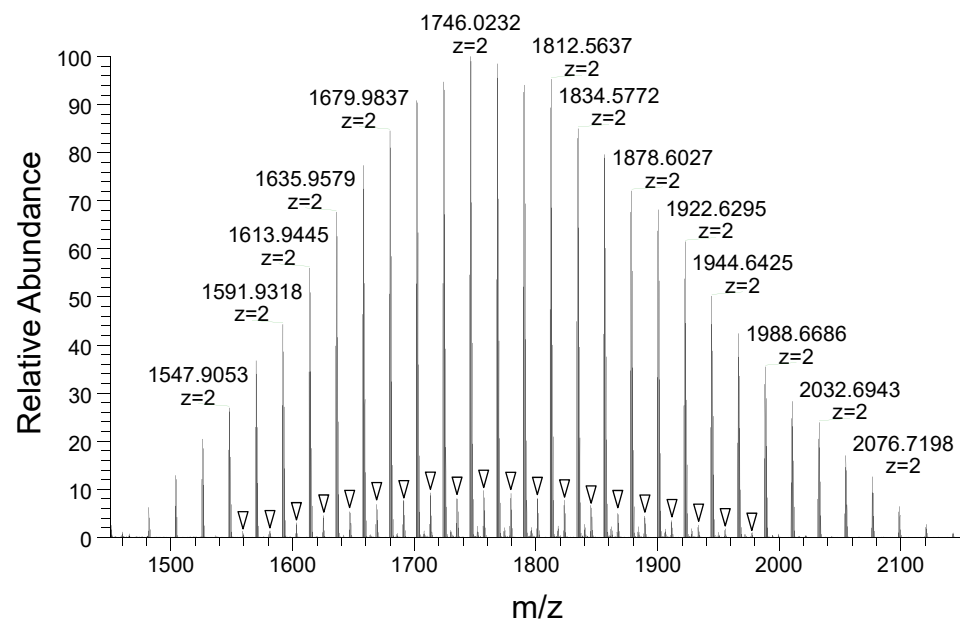

(B)

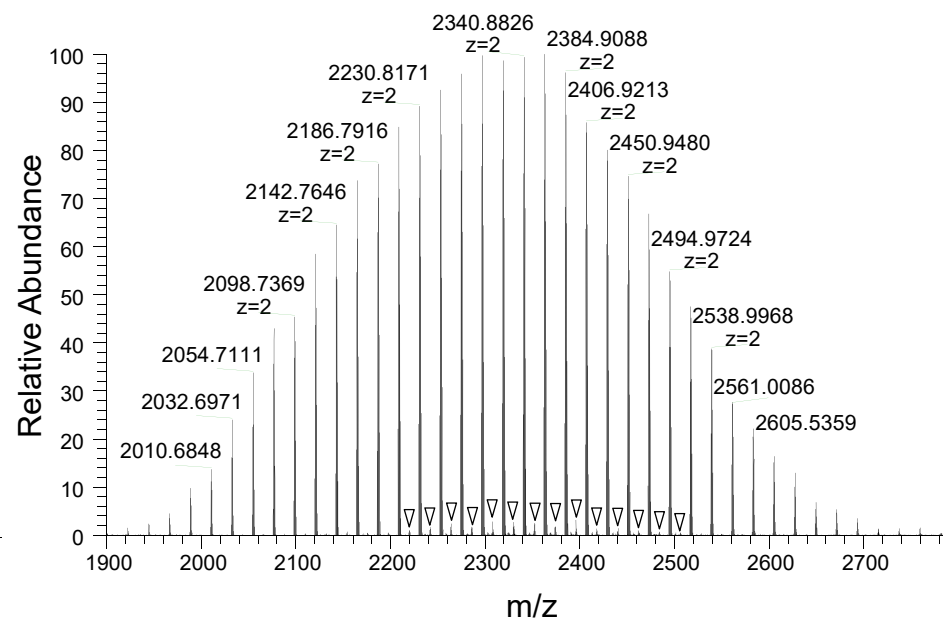

Supplement: S1 Fig — Charge (z) and m/z values are shown for certain signals. The m/z for the series of signals (every 44 Da) was consistent with the m/z value calculated using the structural formula HS-CH2CH2-(OCH2CH2)n-NH2. (A) The major signal series with charge and m/z were assigned to [M + H + Na]2+ ions. The minor signal series with a triangle is [M + 2H]2+. (B) The major signal series with charge and m/z were assigned to the [M + 2Na]2+ ions. The minor signal series with a triangle is [M + H + Na]2+. (PDF) [file pone.0333359.s002.pdf]

**(A)**

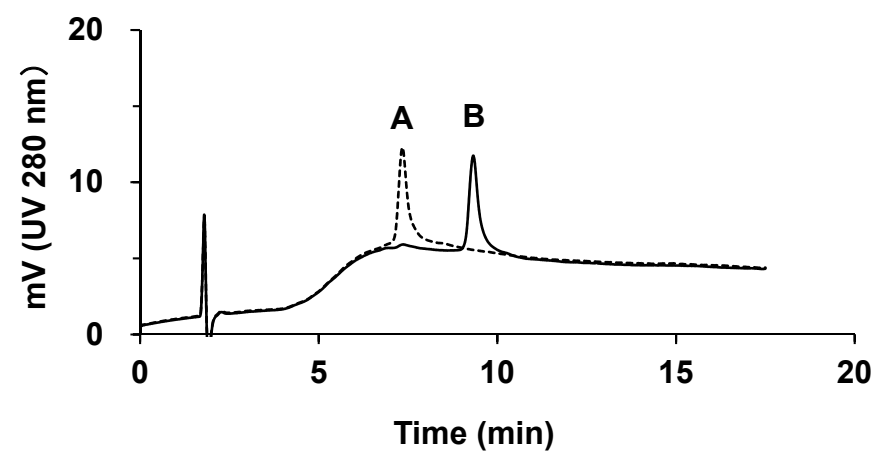

**(B)**

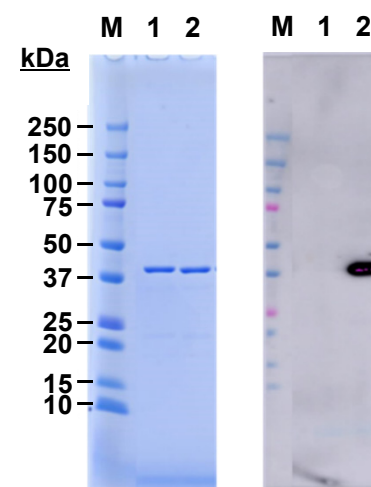

Supplement: S2 Fig — (A) HIC-HPLC profiles of Fabp24 modified with (solid line) or without (dotted line) pentylamine-biotin by MTGase. (B) Non-reducing SDS-PAGE and Western blot image of Fabp24 modified with or without pentylamine-biotin by MTGase. Non-reducing SDS-PAGE and subsequent Western blot analysis were performed following standard procedure. [11] Pentylamine-biotin was detected with HRP-conjugated streptavidin. M, molecular weight marker; 1, Fabp24; 2, Purified product of MTGase-modified Fabp24 with pentylamine-biotin (peak B). (PDF) [file pone.0333359.s003.pdf]

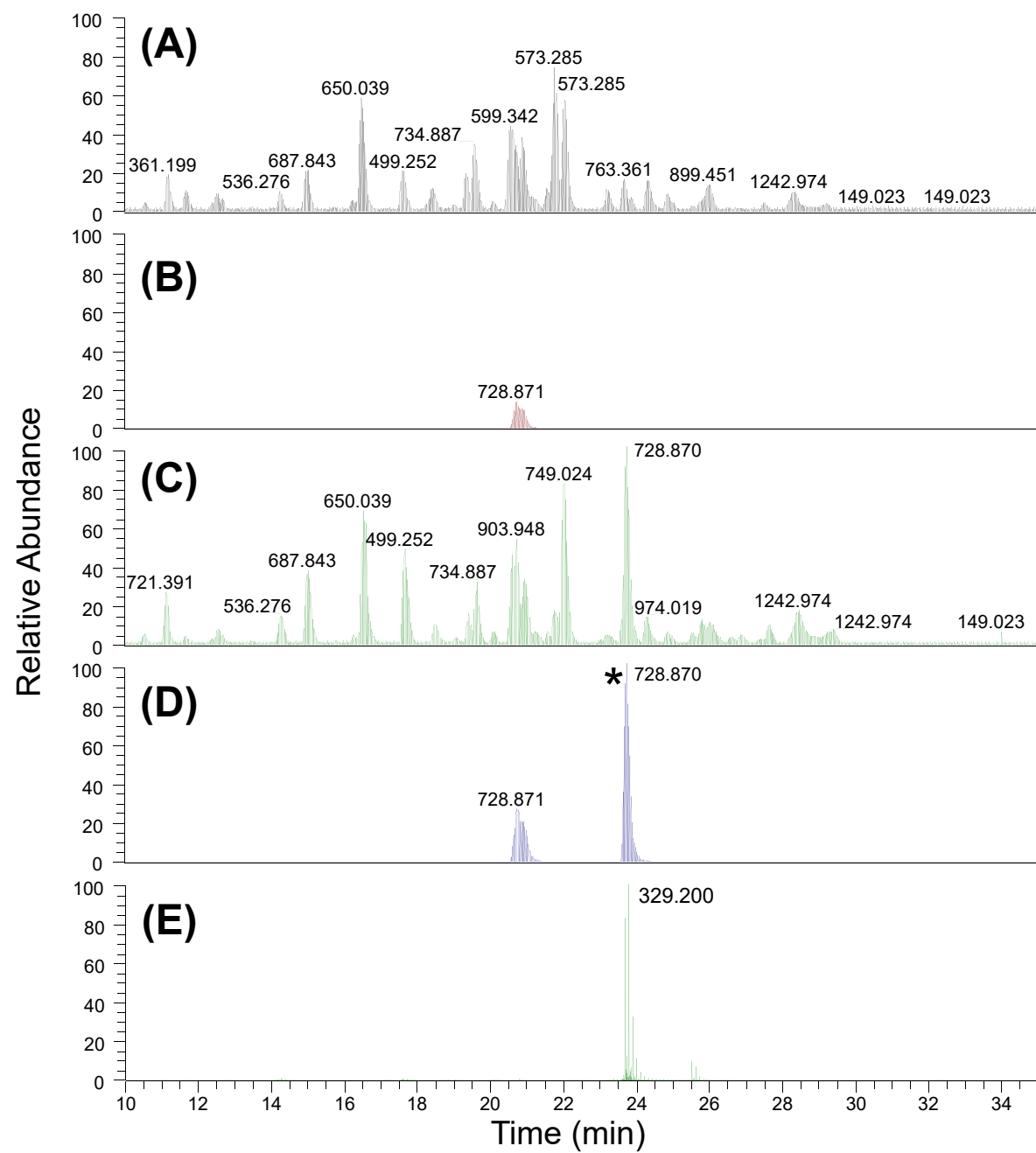

Supplement: S3 Fig — Fabp24 conjugated, with or without pentylamine-biotin modification, was carbamoylmethylated, digested with trypsin, and analyzed using LC-MS and MS/MS. For (A) and (B), unmodified Fabp24 was used, and for (C), (D), and (E), pentylamine-biotin-modified Fabp24 was used. (A) and (C) show base peak intensity chromatograms; (B) and (D) display extracted ion chromatograms of the precursor ion at m/z 728.871 (±20 ppm). (E) presents the extracted ion chromatogram of the product ion at m/z 329.200 (±20 ppm). The asterisk in (D) denotes the target for MS/MS collision-induced dissociation analysis, as shown in Table 1. The extracted ion chromatogram in (E) at m/z 329.200 specifically depicts peptides modified by transglutaminase. (PDF) [file pone.0333359.s004.pdf]

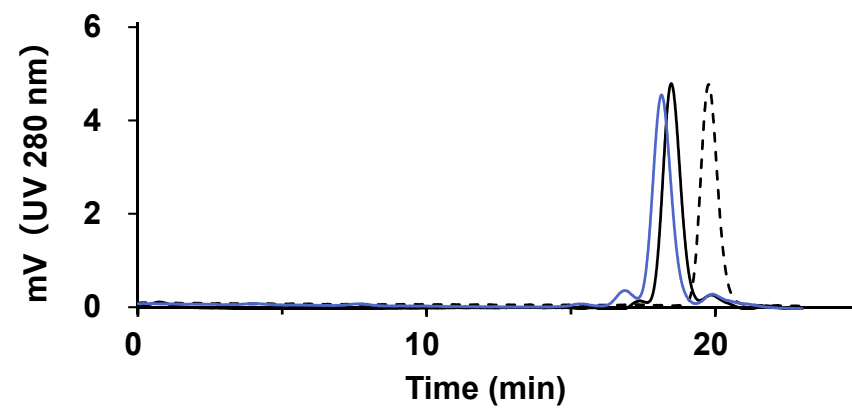

Supplement: S4 Fig — Black dotted line, Fabp24 and MTGase reacted without the HS-PEG linker; black solid line, Fabp24 and MTGase with HS-PEG3.5k; blue line, Fabp24 and M TGase reacted with HS-PEG5k. (PDF) [file pone.0333359.s006.pdf]

(A)

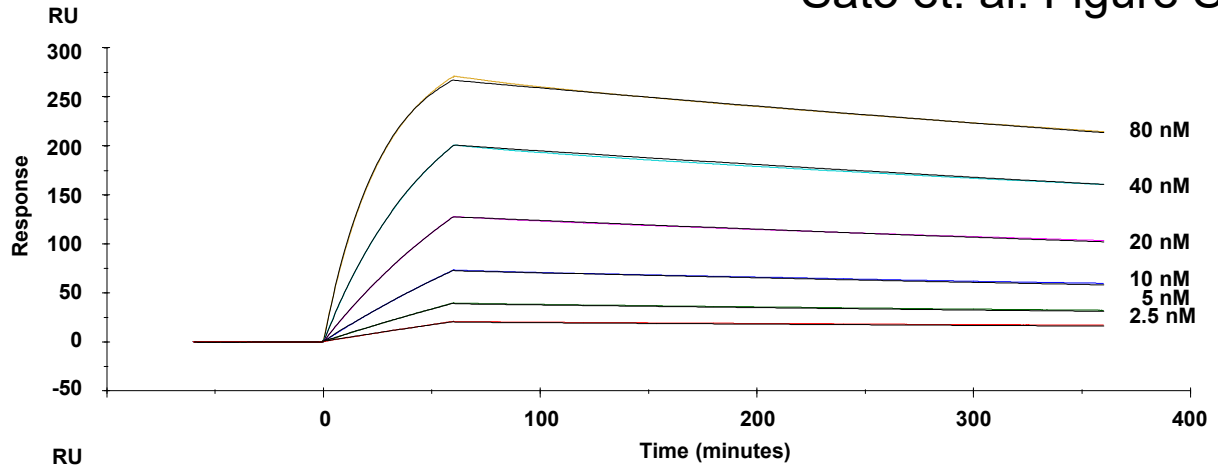

(B)

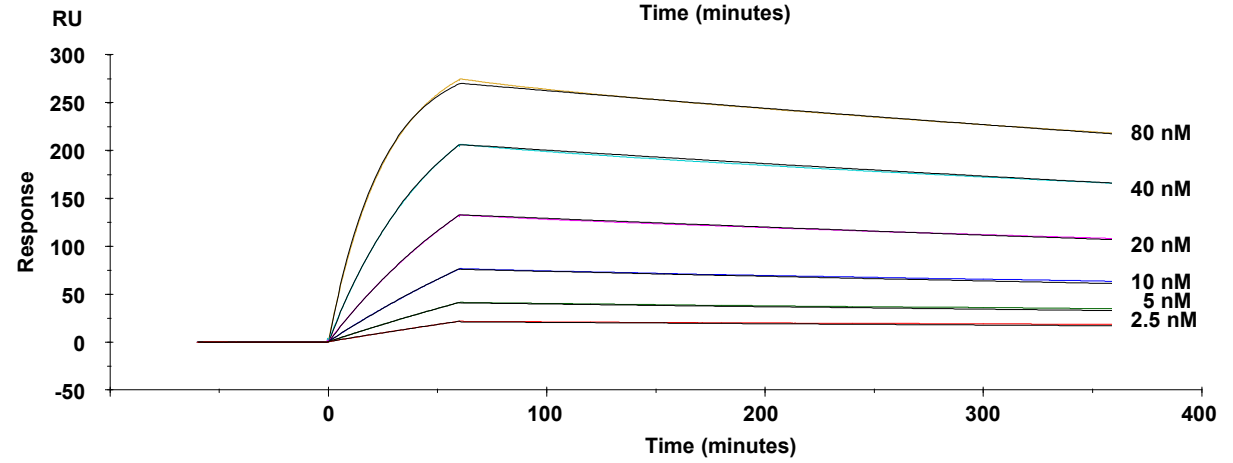

(C)

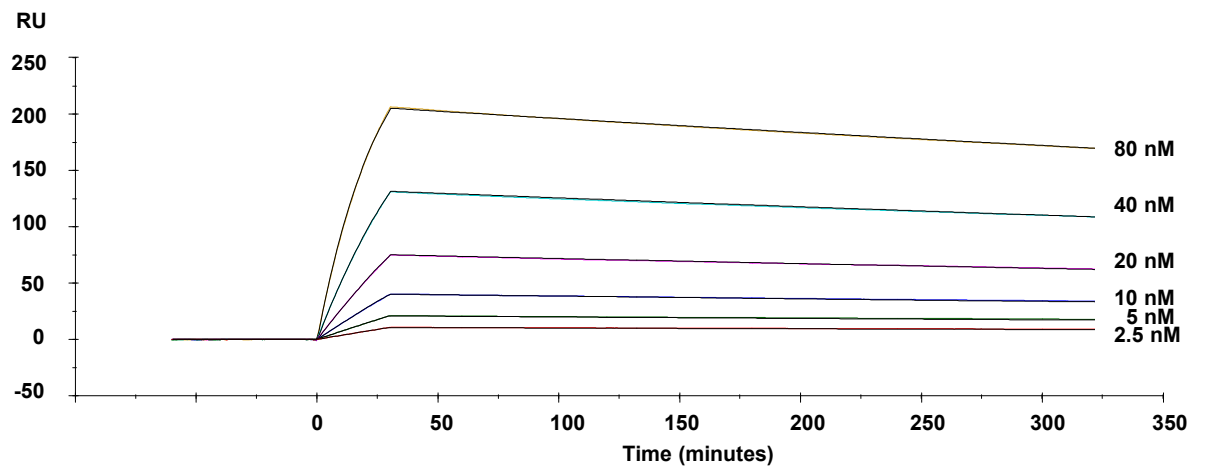

(D)

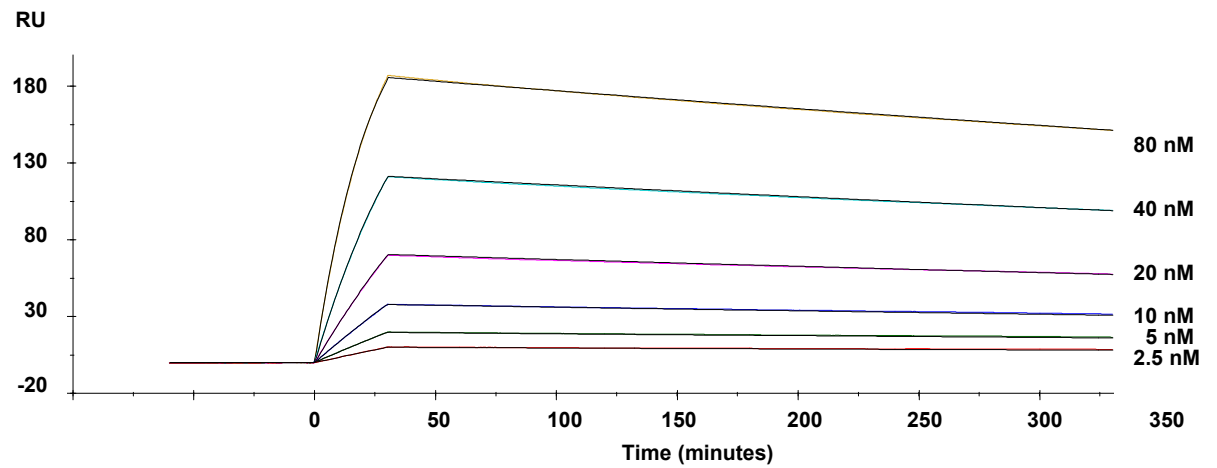

Supplement: S5 Fig — (PDF) [file pone.0333359.s007.pdf]

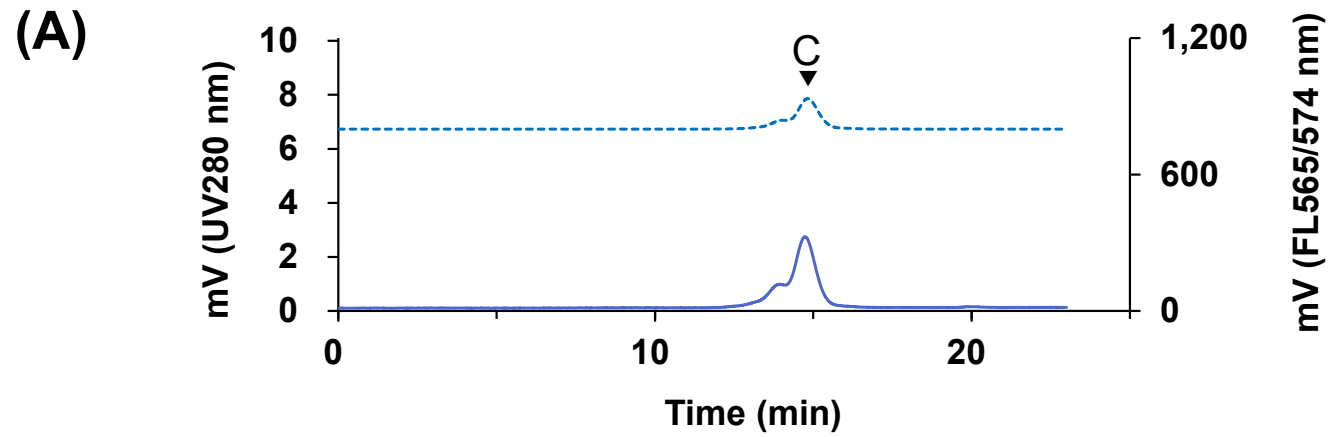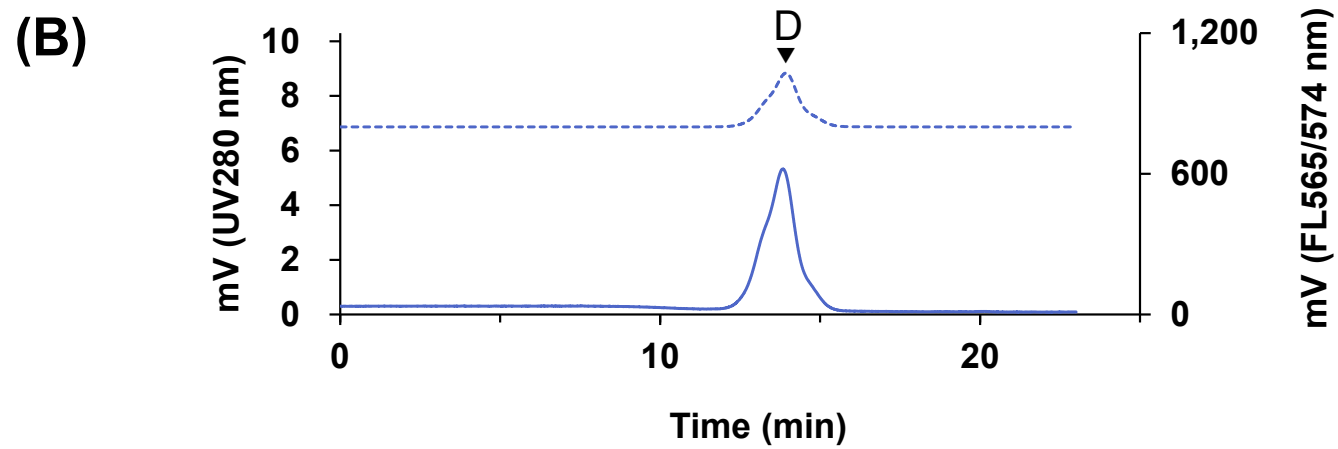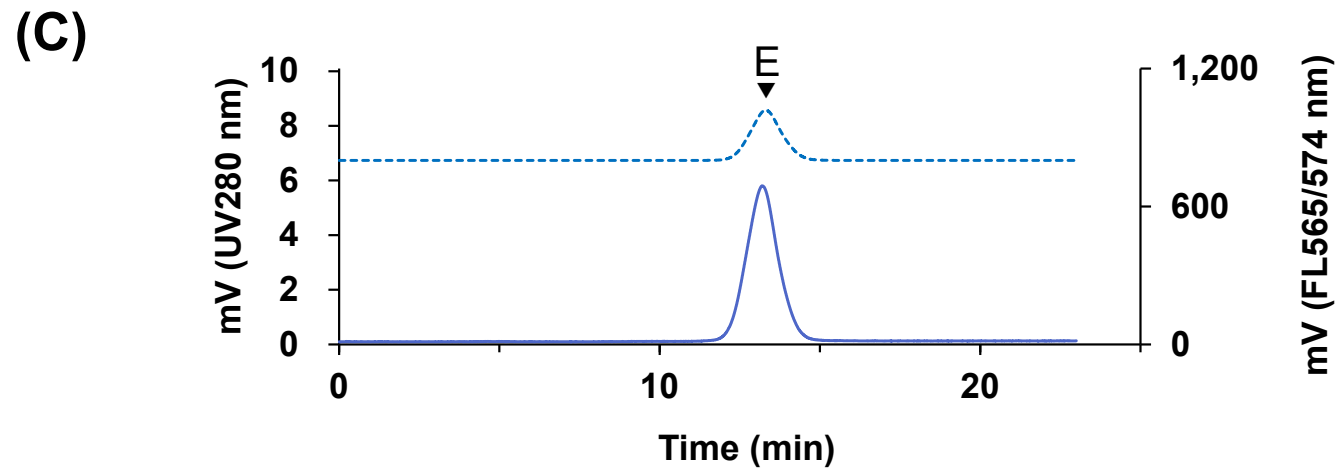

Supplement: S6 Fig — The reaction products of HS-PEG2k-Fabp24 and maleimide-activated PE were fractionated using preparative SEC and analyzed using analytical SEC. Products C–E correspond to those shown in Figure 5. UV: solid line; fluorescence: dotted line. (PDF) [file pone.0333359.s008.pdf]

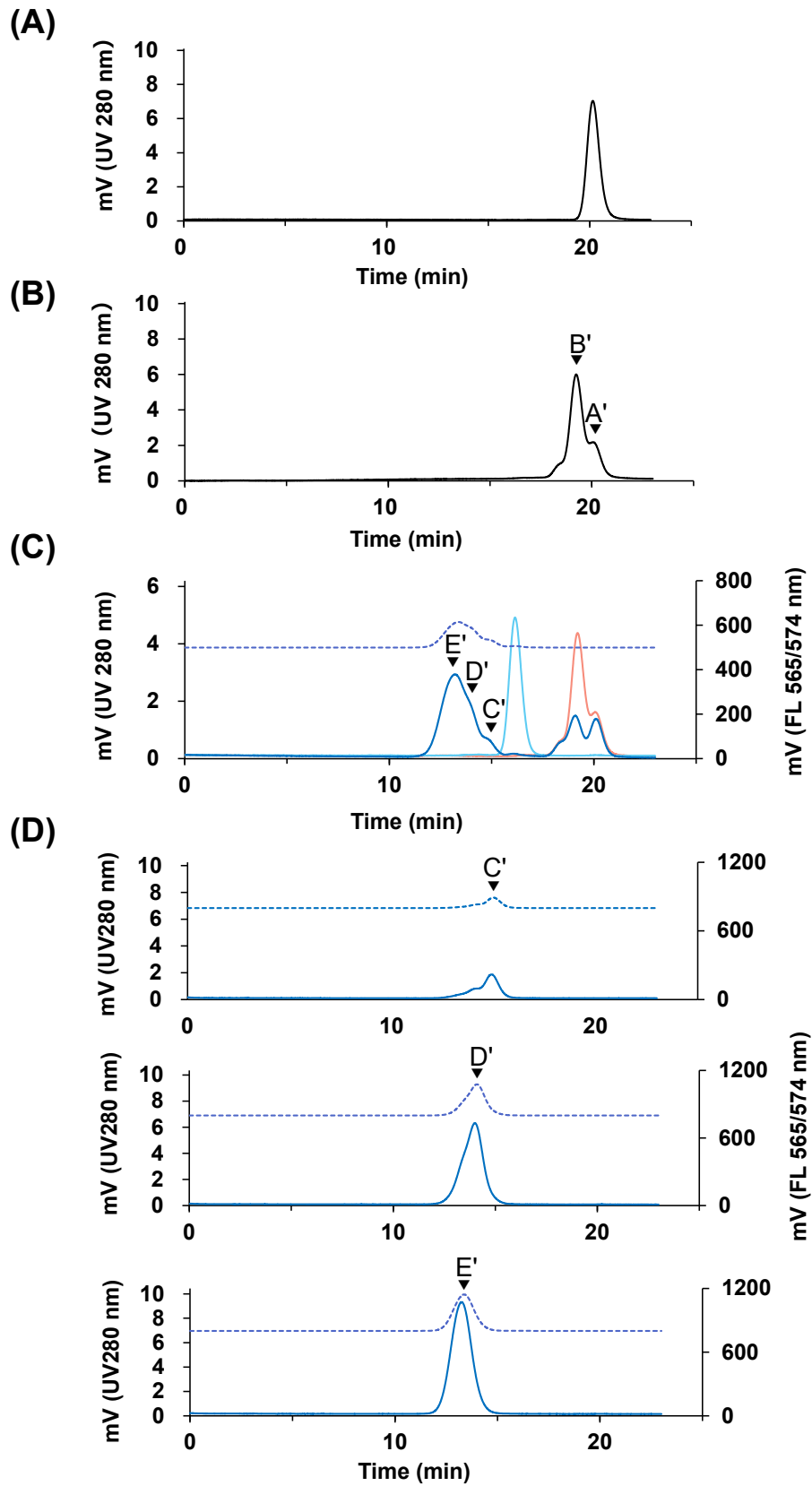

Supplement: S7 Fig — (A) SEC-HPLC chromatogram of FabCD20. (B) SEC-HPLC chromatogram of HS- PEG 2k -Fab CD20. (C) SEC-HPLC chromatograms of the coupling products of HS- PEG-modified FabCD20 with maleimide-activated PE . The reaction products of HS- PEG 2k -Fab CD20 and maleimide-activated PE were analyzed via analytical SEC. The main products forming the peaks are marked with C ' –E ' and arrowheads. UV, solid line; fluorescence, dotted line; reaction product, blue; maleimide-activated PE, sky blue ; HS- PEG 2k -Fab CD20 , orange . (D) SEC-HPLC chromatogram s of the products C ' , D ', and E ' of HS- PEG 2k -Fab CD20 reacted with maleimide-activated PE. The reaction products of HS- PEG 2k -Fab CD20 and maleimide-activated PE were fractionated via the preparative SEC and analyzed by analytical SEC. Products C ' – E ' correspond to those of Figure S7(C) . UV, solid line; fluorescence, dotted line. (PDF) [file pone.0333359.s009.pdf]
