# Supplementary material for: Efficient two-step chemoenzymatic conjugation of antibody fragments with reporter compounds by a specific thiol-PEG-amine Linker, HS-PEG-NH2
Source: PLoS One. 2025 Oct 23;20(10):e0333359. doi: 10.1371/journal.pone.0333359 (PMC12548897; doi:10.1371/journal.pone.0333359)
Supplement: S1 Table — (PDF) [file pone.0333359.s005.pdf]

	**Observed sequence-specific ions from H-S-C-G-V-L-N-L-A-Q-S-P**	**Observed Mass (Da)**	**Calculated Mass (Da)**	**Mass error (ppm)**
**y1**	P	116.071	116.071	3.403
**y2**	S-P	203.113	203.103	1.805
**b2**	H-S-C	248.070	248.070	0.188
**b3**	H-S-C-G	305.091	305.091	-1.367
**b4**	H-S-C-G-V	404.159	404.159	-2.056
**b5**	H-S-C-G-V-L	517.242	517.242	-3.664
**b6**	H-S-C-G-V-L-N	631.284	631.287	-4.471
**b7**	H-S-C-G-V-L-N-L	744.368	744.368	-3.878
**b8**	H-S-C-G-V-L-N-L-A	815.406	814.408	-2.453
